# The Effect of Cannabidiol on Performance and Post-Load Recovery among Healthy and Physically Active Individuals: A Systematic Review

**DOI:** 10.3390/nu16172840

**Published:** 2024-08-24

**Authors:** Eduard Bezuglov, Evgeniy Achkasov, Elizaveta Rudiakova, Vladimir Shurygin, Georgiy Malyakin, Danila Svistunov, Mikhail Butovskiy, Aleksandra Fedorova, Elizaveta Kapralova

**Affiliations:** 1 Department of Sports Medicine and Medical Rehabilitation, Sechenov First Moscow State Medical University of the Ministry of Health of the Russian Federation, 119991 Moscow, Russia; e.n.bezuglov@gmail.com (E.B.); achkasov_e_e@staff.sechenov.ru (E.A.); doc.rudyakova@gmail.com (E.R.); vovashurygin04052001v@mail.ru (V.S.); malyakin_g_i@staff.sechenov.ru (G.M.); dan.sv2012@yandex.ru (D.S.); 2 High Performance Sports Laboratory, Sechenov First Moscow State Medical University, 119991 Moscow, Russia; 3 Department of Rehabilitation and Sports Medicine, Kazan State Medical University of the Ministry of Health of the Russian Federation, 420012 Kazan, Russia; drmike81@inbox.ru; 4 Joint Stock Company «Medsi Group», 123056 Moscow, Russia; fedorova.ala@medsigroup.ru

**Keywords:** cannabidiol (CBD), supplement, athletes, recovery, performance

## Abstract

Athlete performance and post-load recovery can be considered one of the most important and actively discussed topics in professional sport. One substance aimed at improving performance is cannabidiol (CBD), which has been actively gaining popularity with several studies published in recent years. The PubMed, Scopus, and Cochrane Library databases were searched from inception to April 2024 according to PRISMA recommendations to identify studies on the effects of CBD on exercise capacity and post-load recovery. An initial search identified 901 publications, of which seven fully met the inclusion criteria. Current evidence supports a limited beneficial effect of CBD on a number of physiological parameters, such as VO2, mean power, and relative mean power. At the same time, there were limited data on the beneficial effects of CBD on strength parameters (including vertical jump, counter movement jump, one repetition max bench press, and barbell back squat) and post-load recovery. Notably, most of the studies included in the analysis were conducted between 2021 and 2024, indicating a growing interest among researchers in the use of CBD in healthy, physically active individuals. Further studies are needed to assess the safety of different CBD administration protocols in professional athletes.

## 1. Introduction

Improving performance and optimizing post-load recovery can be considered one of the most relevant subjects in professional sports. In this regard, the use of dietary supplements and medicinal substances by professional athletes and the use of various non-pharmacological tools and methods is very common [1,2,3,4,5]. However, good tolerability and safety of these supplements, aids, and methods are very important, especially in the context of strict compliance requirements within the anti-doping legislation [6]. The reduction of anxiety level and pain syndrome severity, as well as the optimization of sleep quality and muscle tissue regeneration, have been proven to be important factors for professional athletes, positively influencing their sporting success [7,8,9]. Recently, the positive effects have been demonstrated by the course or single ingestion of cannabis, used in capsule, oil, and even inhalation forms [10,11]. However, an important limiting factor is the prohibition of its use during the competition period [12]. Moreover, in many countries, the distribution and possession of cannabis is a criminal act. This is due to the tetrahydrocannabinol content of cannabis, which is responsible for the development of various psychoactive effects that make it a recreational drug [13,14]. Another component of cannabis, cannabidiol (CBD), is non-psychotropic and may have beneficial effects on various aspects of health within the general population, including anxiety, sleep quality, and mood [15,16,17,18]. However, cannabidiol, which is not prohibited for athletes, is used by them quite frequently. Therefore, objectifying its effects on parameters important for this sample may be important for choosing an optimal training strategy.

In recent years, there has been a dramatic increase in interest in CBD. For instance, out of 6635 articles that fall under the “cannabidiol” search query on the PubMed database, 5444 come from the period of 2014 to 2024 (status as of April 2024). Together with the removal of CBD in 2018 from the World Anti-Doping Agency’s banned list, this shows an increasing popularity and acceptance of this substance over the last decade [19]. It is worth noting that there are currently two methods of making CBD, the synthetic form and the organic form. For the organic form, it is important to note that if it is insufficiently purified, users may encounter problems with both in-competition doping controls and legislation [20,21].

Considering the described effects, CBD can be seen as one of the potential aids to improve the performance and post-load recovery of physically active individuals. Nevertheless, there is no systematic review on this topic, therefore it is of practical interest to conduct one.

The main objective of the current study was to objectify the possible effects of cannabidiol supplementation on exercise performance and exercise tolerance in physically active individuals and to analyze the possible limitations of previous studies.

## 2. Methods

The PubMed, Scopus, and Cochrane Library databases were searched from their inception to April 2024 in accordance with PRISMA recommendations to identify studies on the effects of CBD on exercise capacity and post-load recovery in healthy, physically active people [22]. For this purpose, the following search query has been used: (cannabidiol OR CBD) AND (performance OR “maximal aerobic power” OR “physiological functions” OR “physical indicators” OR “comparative physiology” OR loads OR endurance OR strength OR speed OR coordination OR recovery OR doping OR “peak load” OR exercise OR “physical activity” OR “physical loading” OR “exercise stress” OR regeneration OR “VO2” OR “VO2Max” OR efficiency OR fatigue OR sleep OR overwork OR “muscle pain” OR pain OR “delayed onset muscle soreness” OR “DOMS”) AND (athletes OR sport OR training OR “physical exercises” OR fitness OR “elite athlete” OR competitions).

The search results were downloaded and filtered in the Mendeley Reference Manager v2.64.0 (Mendeley Ltd., London, UK) systematic review software. The PICOS design framework was used to screen the titles and abstracts of retrieved articles [23], as follows:

«P» (population): studies involving healthy, physically active people with no restrictions on gender, age, or fitness level;

«I» (intervention): any administration route of the substance “cannabidiol” for participants in the main group;

«C» (comparison): participants who received the substance “cannabidiol” during this study were compared with those who received a placebo;

«O» (outcome): various changes in performance, post-load recovery, and fatigue levels were analyzed;

«S» (study design): original randomized controlled trials (including those with a crossover design) conducted in humans were included in the review.

The initial literature screening resulted in an initial sample of studies covering various aspects of CBD intervention. Studies were analyzed in detail, taking into account study design, participant characteristics, methods of measuring physical performance, CBD dosages, duration of experiments, and other relevant factors. Studies were categorized according to the type of sports activity and results. After analyzing the publications, the results of different studies were compared, general trends were identified, and conclusions about the effects of CBD on physical performance and post-load recovery in healthy, physically active individuals were formulated.

All identified studies were assessed by 3 authors for risk of bias employing the revised Cochrane Risk of Bias Tool for Randomized Trials (RoB 2) (Cochrane, Bristol, UK). Cases of disagreement between researchers were resolved through discussion or consultation with the senior investigator.

## 3. Results

**Figure 1:**
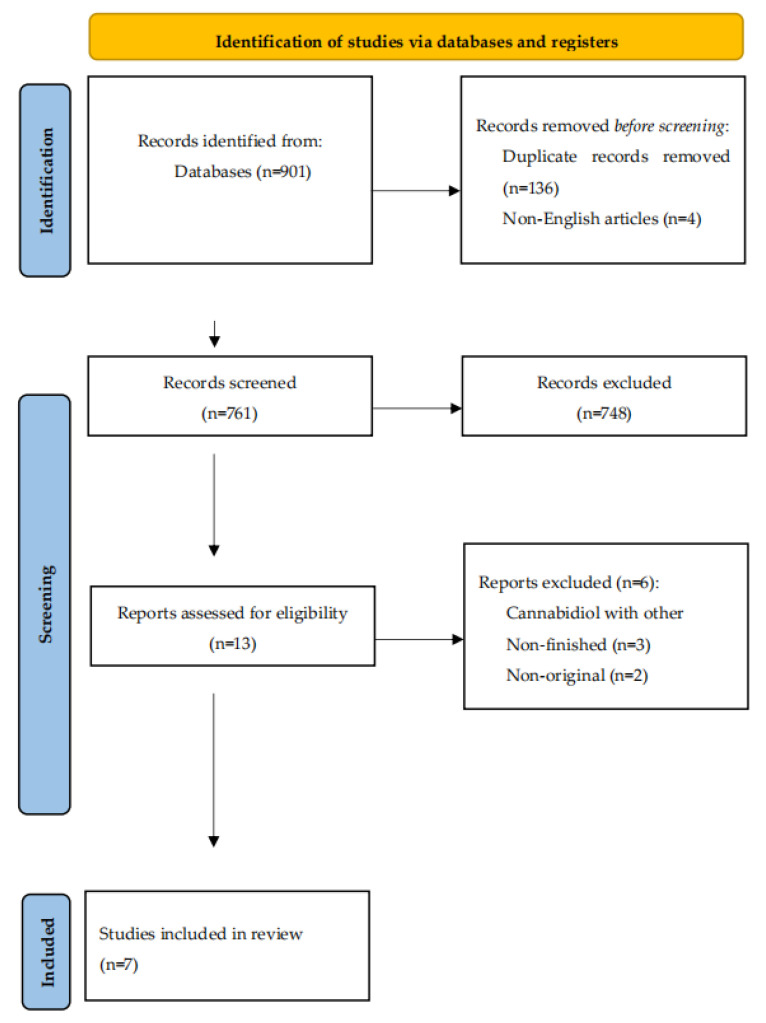
Flowchart identifying the process to select studies.

A total of 901 articles were selected from the analyzed databases based on keywords and their combinations; 140 articles were excluded (136 duplicates, 4 in non-English). After the analysis of the titles and abstracts by three authors independently, 748 of the remaining 761 articles were excluded, as they did not meet the main criteria and subject of this systematic review. Six of the remaining thirteen articles were removed after careful screening, one of which used CBD with a combination of other substances as an intervention, three did not present research results (incomplete study), and two papers presented only abstracts (Figure 1).

Thus, seven publications (134 participants) were found in English describing the results of studies involving healthy, physically active individuals that evaluated the effects of CBD on performance and post-load recovery. In addition, the reference list of each of the seven articles, as well as other publications by all co-authors, was examined to minimize the risk of missing articles in this systematic review (Table 1).

It should also be noted that all but one of the studies included in this review can be classified as studies of high methodological quality; they were all randomized with a sufficient sample size and placebo use. Only one study raised doubts about the validity of the results due to the small sample size (4 people), a limitation that was also pointed out by the authors of this study [30] (Table 2).

## 4. Discussion

This review demonstrates that interest in the use of CBD among healthy, physically active people has increased in recent years—the results of all identified studies were published between 2021 and 2024. This is most likely due to the previously available data on the positive effects of CBD on anxiety, sleep disorders, and chronic pain in the general population [31,32,33].

The exact mechanisms of how CBD works are unknown. However, there are several assumptions about how it works. There are a large number of potential receptors in the human body with which it can interact and exert a fairly wide range of effects. For example, interaction with CB2, TRPV1, and PPARγ, as well as adenosine receptors, may have an anti-inflammatory effect. Interaction with 5-HT1A and TRPV1 receptors may reduce pain syndromes, including chronic pain. Interaction with the 5-HT1A receptors is also associated with anxiolytic effects [34]. The potential mechanisms of action of the CBD are described in more detail in the following works [35,36].

To date, there is still a lack of such studies, especially those involving the regular administration of CBD; only one such study has been conducted, in which CBD was administered for 8 weeks at 50 mg daily [27]. The absence of a uniform protocol for the administration of CBD and the heterogeneity of its dosage forms used in the studies is another important issue. Three of them used CBD capsules [24,27,30], one used oil or capsules pre-dissolved in water [28], one used only capsules pre-dissolved in water [26], and two used only oil [25,29]. However, the heterogeneity of the forms used could probably be neglected, as no statistically significant differences were reported with respect to peak concentration, time to peak concentration, and effects on cardiovascular, hepatic, and renal function in studies investigating differences in the pharmacokinetics of the two forms of CBD release (oil and capsules) [37].

Importantly, the studies included in this systematic review used both organic [24,27,29,30] and synthetic [25] forms of CBD. In two other studies, the form of CBD used was not specified [26,28].

Importantly, none of the studies reported any adverse effects of CBD administration, suggesting a favorable safety profile. This aspect is particularly beneficial for athletes, as it allows them to potentially enhance their performance without the risk of negative side effects, which is a crucial consideration in the context of competitive sports.

In all but one study [29], limited positive effects of different forms of CBD (capsules, oil, and capsules pre-dissolved in water) on a range of physiological parameters important for physical performance (VO2max, average power) were observed. It was demonstrated that serum levels of myoglobin, lactate, and CK were statistically significantly higher in CBD supplementation groups compared to placebo groups [24,25,26]. This may be due to the increased performance of the participants who consumed CBD and, as a consequence, higher serum CK and myoglobin levels, which are associated with more intense training and competition loads [38,39].

Other studies have demonstrated statistically significant improvements in VO2 max, average power, and relative average power among participants using CBD compared to participants in control groups [25,27].

The administration of CBD may also be associated with limited positive effects on fatigue levels and psycho-emotional states during and after exercise [24,25].

At the same time, the studies concerned with using CBD to evaluate strength parameters are equivocal; no positive effects of CBD in these studies have been obtained on strength parameters important for professional athletes (vertical jump, countermovement jump, one repetition of maximal bench press, one repetition of maximal barbell back squat, dynamic and isometric strength measurements), which should be taken into account in future studies. None of the studies showed statistically significant improvements in these tests [26,27,28].

In one study [30], two groups were compared to a placebo group at once. The first group took a low dose of CBD (2 mg/kg), and the second took a high dose (10 mg/kg). The effect of CBD on such parameters as IL-6 level was studied (after 24 h its increase was noted in both groups and after 48 h only in the high dose group), as well as handgrip (after 24 and 48 h after administration, the increase in values was noted only in the low dose group, after 72 h, in both groups), bicep curl (after 48 and 72 h, increase in values was noted in both groups), and pain on VAS scale (pain sensations became more intense in both groups after 24, 48, and 72 h), which may be related to more intensive work during test exercises against the background of increased performance from CBD intake. However, due to the small sample size, the results of this study should be interpreted with caution. Moreover, this limitation was pointed out by the authors themselves.

## 5. Limitations

This systematic review has limitations related to the small sample size, inconsistent outcome measures, lack of statistical synthesis, and potential biases that undermine the reliability and validity of the findings. Future research should aim to address these limitations by including larger and more diverse samples, standardizing outcome measures, and conducting rigorous statistical analyses.

A limitation of the studies, from a professional sports context, is the participation of physically active members of the general population rather than athletes. Another limitation is the lack of testing protocols that assess sport-specific qualities and skills.

Therefore, future research should focus on the effects of CBD on the sport-specific skills of professional athletes. These studies should examine the effects of different dosing regimens of different forms of CBD and thoroughly assess the safety spectrum.

## 6. Conclusions

Current evidence supports a limited positive effect of CBD on a range of physiological parameters related to physical performance, but not post-load recovery. Further research should utilize different protocols of CBD administration to assess the safety spectrum among professional athletes.

## Figures and Tables

**Table 1:** Details and results of the studies investigating the effects of cannabidiol on various measures in healthy people with active lifestyles.

Study	Design	Population	Outcome Analyzed	Intervention	Results
Crossland et al., 2022 [24]	RCT crossover	24 well-trained females; age = 21.2 ± 1.8 years; height = 166.4 ± 8 cm; weight = 64.9 ± 9.1 kg	MB, inflammatory markers (IL-10, IL-1β, IL-6), dynamic strength, isometric strength, VJ, peak torque, peak isometric torque, VAFS	1st, 4th visits: Blood sampling; CBD/placebo capsules administration, exercise (eccentric leg extensions). 2nd, 3rd, 5th, 6th visits: performance measurement: blood draw; VJ, isometric and dynamic strength analysis, VAFS	MB ↑. Inflammatory markers=. Dynamic strength=. Isometric strength=. VJ=. After 4–24 h after training: peak torque ↓, peak isometric torque ↓. VAFS ↑.
Sahinovic et al., 2022 [25]	RCT	9 endurance-trained males: 18–45 years, running average ≥40 km/wk. Weight: PLA = 70.5 ± 5.4 kg, CBD = 70.7 ± 5.5 kg	VO2, VO2max, blood glucose, lactate, RPE, rating of pleasure, TTE, AEA, HR, HRmax, RERmax	CBD (300 mg)/Placebo capsules administration 90 min before RUN 1: 60 min at an intensity of 70% VO2max, 30 min of rest. RUN 2: increasing by 2% every 3 min until volitional exhaustion	RUN 1: VO2 ↑, rate of pleasure ↑, lactate ↑. HR=, RPE=, blood glucose=, RER=. RUN 2: VO2max ↑, RERmax ↑, TTE=, HRmax=. Post RUN 1 and RUN 2: AEA ↑. Post- RUN 2: AEA ↓.
Isenmann et al., 2021 [26]	RCT crossover	16 experienced (at least one year) in strength training age = 24 ± 3 years; height = 181.4 ± 10.0 cm; weight = 79.2 ± 13.7 kg	MB, CK, 1 RM BS, CMJ	First visit: anthropometry + blood sample. Warm-up; Maximal power and strength test (before and after 24, 48, 72 h training). Training protocol: 3 × 12 BS 70% of their 1RM with 150 s rest between sets. Drop jumps from a 45 cm high box, landed in a deep squat, 3 × 15 with 60 s rest between sets. 60 mg CBD solubilisat with 250 mL water/PLA drink directly after exercise.	After 24 h: 1 RM BS ↓, CK ↑, MB ↑, CMJ=. After 48 h: CK ↑, MB ↑, 1 RM BS=, CMJ=. After 72 h: CK ↑, MB ↑, 1 RM BS=, CMJ=.
Flores et al., 2023 [27]	RCT	48 physical active participants (24 males and 24 females). Age = 25 ± 6 years; height = 171 ± 10 cm; weight 73 ± 13 kg	Lean body mass, Body fat percentage, VO2max, 1 RM BP, 1RM BS, mean power, relative mean power, anaerobic fatigue, steps per day, psychological wellbeing, CRP	8 visits: 4 pre-interventions (blood sampling, psychological wellbeing, anthropometry, aerobic tests, anaerobic test, muscle strength test) and 4 after. During the 8-week intervention, participants consumed 50 mg of hemp-derived CBD/225 mg of a placebo capsules.	Lean body mass=, Body fat percentage=, VO2max=, 1 RM BP=, 1 RM BS=, mean power ↑, relative mean power ↑, anaerobic fatigue=, steps per day=, psychological wellbeing=, CRP=.
Isenmann et al., 2024 [28]	RCT crossover	17 well-trained athletes of an advanced strength level (15 males and 2 females)	CMJ, BS, BP, 1-mile run, CK, MB, IL-6, IL-10, oxidative stress markers, immune cell activity markers	3 × 6 days high-intensity training protocol. After training: CBD/placebo oil (60 mg). Between the intervention phases, at least 4 weeks of washout period.	CBD Oil-AD: CMJ=; 1RM BS=; 1RM BP=; 1mile=; CK=; MB ↓; IL-6=; IL-10=; OxLDL=; ImAnOx=; PLR trend ↑; NLR=; SII=. CBD solu-AD: CMJ=; 1RMBS=; 1RMBP=; 1mile=; CK=; MB=; IL-6=; IL-10=; OxLDL=; ImAnOx=; PLR=; NLR=; SII=. CBD Oil-Hi: CMJ=; 1RMBS=; 1RMBP=; 1mile=; CK=; MB=; IL-6=; IL-10=; OxLDL=; ImAnOx=; PLR trend ↑; NLR=; SII=. CBD solu-Hi: CMJ ↑; 1RMBS=; 1RMBP=; 1mile=; CK ↑; MB ↑; IL-6=; IL-10=; OxLDL strong trend ↑; ImAnOx=; PLR=; NLR=; SII=.
Skopek et al., 2021 [29]	RCT crossover	16 healthy males in good physical shape. Age: 22.7 ± 0.8 years; height 178.1 ± 8.6 cm; weight 75.7 ± 6 kg	SRT; CRT	The experiment was conducted twice at one-week intervals. The participants were administered CBD (11 mg)/PLA oil sublingually. 30 min after—was tested with a reactometer for SRT and CRT.	SRT=, CRT=.
Stone et al., 2023 [30]	RCT, double-blind, crossover	4 physically active participants (2 males and 2 females). Height 171.38 ± 9.60 cm; weight 71.89 ± 16.30 kg; body composition 22.82 ± 7.44%.	Pain, Range of Motion, IL-6, Handgrip, Bicep Curl, Determination	Supplementation/PLA capsules: 0 h and 12 h after 2 mg/kg or 10 mg/kg. Baseline measurements, 24 h, 48 h, 72 h	IL-6: After 24 h: low ↑, high ↑; After 48 h: low ↓, high ↑; After 72 h: low ↓, high ↓. Handgrip: After 24 h: low ↑, high ↓; After 48 h: low ↑, high ↓; After 72 h: low ↑, high ↑. Bicep Curl: After 24 h: low ↓, high ↓; After 48 h: low ↑, high ↓; After 72 h: low ↑, high ↓. Pain: After 24 h: low ↑, high ↑; After 48 h: low ↑, high ↑; After 72 h: low ↑, high ↑. Range of Motion: After 24 h: low ↓, high ↓; After 48 h: low ↓, high ↓; After 72 h: low ↓, high ↓.

1RM—one repetition maximum; AD—advanced; AEA—anandamide; BP—bench press; BS—back squat; CBD—cannabidiol; CK—creatine kinase; CMJ—countermovement jump; CRP—C-reactive protein; CRT—complex reaction time; Hi—highly advanced; high—high doses of CBD (10 mg/kg); HR—heart rate; HRmax—maximal heart rate; IL—interleukin; ImAnOx—Antioxidative Capacity; low—low doses of CBD (2 mg/kg); MB—myoglobin; N/E—not evaluated; NLR—neutrophil granulocyte/lymphocyte ratio; OxLDL—Oxidized Low Density Lipoprotein; PLA—placebo; PLR—platelet-to-lymphocyte-ratio; RCT—randomized controlled trial; RER—respiratory exchange ratio; RERmax—maximal respiratory exchange ratio; RPE—rate of perceived exertion; SII—systemic immune-inflammation index; SRT—simple reaction time; TTE—time to exhaustion; VAFS—visual analog fatigue scale; VJ—vertical jump; VO2—oxygen volume; VO2max—maximal oxygen volume; ↑: Increase in indicators; ↓: Decrease in indicators; =: No effect.

**Table 2:** Result of risk of bias assessment for each randomized control trial included in a systematic review.

Study	D1	D2	D3	D4	D5	D6	D7	D8	Overall
Crossland et al., 2022 [24]	+	+	+	+	+	+	+	+	+
Sahinovic et al., 2022 [25]	+	+	+	+	+	+	+	+	+
Isenmann et al., 2022 [26]	+	+	+	+	+	+	+	+	+
Flores et al., 2023 [27]	+	+	+	+	+	+	+	+	+
Isenmann et al., 2024 [28]	+	+	+	+	+	+	+	+	+
Skopek et al., 2021 [29]	+	+	+	+	+	+	+	+	+
Stone et al., 2023 [30]	+	+	+	−	−	−	+	+	+

Domains: D1: Bias arising from the randomization process. D2: Bias due to deviations from intended intervention. D3: Bias due to missing outcome data. D4: Bias in measurement of the outcome. D5: Bias in selection of the reported result. D6: Conflict of interest bias. D7: Funding sources bias statement. D8: Publication bias. − Some concerns + Low concerns.

## Data Availability

The datasets used and/or analyzed during the current study are available from the corresponding author upon reasonable request.
